# Protein Profiling of Malaria-Derived Extracellular Vesicles Reveals Distinct Subtypes

**DOI:** 10.3390/membranes12040397

**Published:** 2022-04-01

**Authors:** Tosin Opadokun, Jeffrey Agyapong, Petra Rohrbach

**Affiliations:** Institute of Parasitology, McGill University, Sainte-Anne-de-Bellevue, QC H9X 3V9, Canada; veronica.opadokun@mail.mcgill.ca (T.O.); jeffrey.agyapong@mail.mcgill.ca (J.A.)

**Keywords:** *Plasmodium falciparum*, malaria, extracellular vesicles, red blood cells, proteins

## Abstract

Malaria is caused by obligate intracellular parasites belonging to the genus *Plasmodium*. Red blood cells (RBCs) infected with different stages of *Plasmodium* spp. release extracellular vesicles (EVs). Extensive studies have recently shown that these EVs are involved in key aspects of the parasite’s biology and disease pathogenesis. However, they are yet to be fully characterized. The blood stages of *Plasmodium* spp., namely the rings, trophozoites and schizonts, are phenotypically distinct, hence, may induce the release of characteristically different EVs from infected RBCs. To gain insights into the biology and biogenesis of malaria EVs, it is important to characterize their biophysical and biochemical properties. By differential centrifugation, we isolated EVs from in vitro cultures of RBCs infected with different stages of *Plasmodium falciparum*. We performed a preliminary characterization of these EVs and observed that important EV markers were differentially expressed in EVs with different sedimentation properties as well as across EVs released from ring-, trophozoite- or schizont-infected RBCs. Our findings show that RBCs infected with different stages of malaria parasites release EVs with distinct protein expression profiles.

## 1. Introduction

Malaria is a complex human parasitic disease that is caused by five *Plasmodium* species, namely *P. malariae*, *P. ovale*, *P. vivax*, *P. knowlesi* and *P. falciparum*. In 2020, the global estimated number of cases and deaths due to malaria exceeded 240 million and 600,000, respectively, with Africa being disproportionately affected [1]. The bulk of the public health burden due to malaria emanates from *P. falciparum*, the most lethal malaria parasite, and the primary cause of severe malaria. Severe malaria most commonly presents as severe anemia, respiratory distress, multiorgan failure and cerebral malaria, the latter of which is characterized by coma and other neurological complications [2]. Delayed or poor management of severe malaria may result in death.

In humans, infection with malaria occurs following the blood meal of an infected female *Anopheles* mosquito. Shortly after infection, the parasite enters and multiplies in liver cells before invading red blood cells (RBCs). Within RBCs, the parasite enters a cycle of asexual development from rings through trophozoites, to schizonts. These blood stages of the parasite are solely responsible for the clinical manifestations of malaria and hyperparasitemia is a hallmark of severe malaria [2,3]. 

Much remains to be understood about the pathogenesis of severe malaria. The majority of what is known has come from extensive studies of the biology of the parasites’ asexual blood stages and how infected RBCs (iRBCs) directly interact with various host cells, as orchestrated by *P. falciparum* virulence factors [4,5,6,7,8]. More recently, studies have shown that indirect interaction plays a vital role in the pathophysiology of malaria, whereby communication is not only between *P. falciparum* iRBCs and other host cells [9,10,11,12,13,14,15,16] but also between populations of *P. falciparum* [10,17]. These studies have been extensively reviewed [18,19]. The mediators of these alternative means of intercellular communication are called extracellular vesicles.

The term extracellular vesicles (EVs) encompasses membrane-bound particles secreted from viable cells and that originate from endosomes (exosomes) or plasma membranes (microvesicles); they range broadly in size and may be <200 nm (small EVs) or >200 nm (medium or large EVs) [20,21]. EVs are increasingly becoming a focus of study in widely varying fields of research consequent of their diverse biomolecular composition, core roles as intra- and intercellular communicators in pathologic and physiologic conditions and their increasing application as tools in diagnostics, prognostics, and treatment of diseases. The doorway to accessing the full biomedical potential is to characterize the biophysical and biochemical properties of EVs due to their intrinsic heterogeneity [22].

EVs secreted by *P. falciparum*-iRBCs are anticipated to comprise a heterogeneous population of vesicles that originate from the host RBC as well as the parasite. Furthermore, these EVs may differ depending on the intraerythrocytic stage of the parasite since rings, trophozoites and schizonts have distinct morphological, biochemical, and metabolic phenotypes [23,24,25]. Herein lies the peculiar challenge and importance of characterizing malaria derived EVs. While mature RBCs are not known to release exosomes due to the lack of required endosomal machinery, they do shed microvesicles as part of a homeostatic aging process [26,27]. In contrast, recently published data suggests that *P. falciparum* releases both microvesicles and exosomes [10,17,28].

During malaria, only a small proportion of RBCs are infected and may be up to 10% of RBCs in natural human infection [3]. In vitro cultures, though often maintained at 3–5%, can be manipulated to reach much higher parasitemia [29]. With careful consideration to minimize the confounding effect of RBC derived microvesicles on our analysis of *P. falciparum* iRBC derived EVs, we have isolated and characterized EVs from stage-specific *P. falciparum* iRBC cultures to identify core similarities and/or dissimilarities in their EV marker profile. Key factors that were considered in our in vitro culture system and differential centrifugation EV isolation protocol include the age of RBCs, proportion of total RBCs (hematocrit) and parasitized RBCs (parasitemia) in culture, the synchronicity of each life stage (rings/trophozoites/schizonts), as well as the culture media used and the volume of conditioned culture media (CCM) from which EVs were isolated. In accordance with MISEV2018 guidelines [20], analyses of our isolates for important transmembrane (CD63, CD81, and glycophorin A) and cytosolic (Flotillin 2) proteins demonstrated the presence and nature of the EVs. We found these EV markers to be differentially abundant in EVs pelleted at different speeds and across EVs released from the ring-, trophozoite- or schizont-iRBCs.

The characteristically different EVs released from stage-specific *P. falciparum* iRBCs may be implicated in different aspects of parasite biology and disease pathophysiology. It is also plausible these EV subtypes have overlapping functions and applications. Here, we present preliminary findings of the protein profiling of malaria derived EVs. These findings provide a foundation to develop further research in characterizing EVs in malaria infection to delineate their unique profiles and understand malaria EV biology and biogenesis.

## 2. Materials and Methods

### 2.1. Materials

The culture medium was Multicell RPMI 1640, 1× obtained from Wisent Inc.; Saint-Jean-Baptiste, QC, Canada (350-005-CL). Primary antibodies for Western blot analysis were as follows: mouse monoclonal anti-human CD63 (10628D; Invitrogen; Waltham, MA, USA), mouse monoclonal anti-CD81 (10630D; Invitrogen), rabbit monoclonal anti-human glycophorin A (GPA-ab129024; Abcam; Cambridge, UK), rabbit polyclonal anti-*P. falciparum Pf* GRP78 (BiP, MRA-1246; BEI Resources; Northern Virginia, United States), rabbit monoclonal anti-flotillin 2 (ab181988; Abcam), rabbit monoclonal anti-albumin (ab192603; Abcam), rabbit polyclonal anti-hemoglobin (PA5-97488; Invitrogen). HRP-conjugated secondary antibodies purchased from Abcam were goat anti-mouse IgG (ab6789) and goat anti-rabbit (ab97080). Nalgene 0.45 μm pore PES rapid flow vacuum filter units (165-0045) and Pierce silver stain kit (24612) were from ThermoFisher; Waltham, United States. Satorius vivacell 100 PES concentrators were purchased from Fisher Scientific (VC1042). 

### 2.2. Plasmodium falciparum Culture

**Figure 1:**
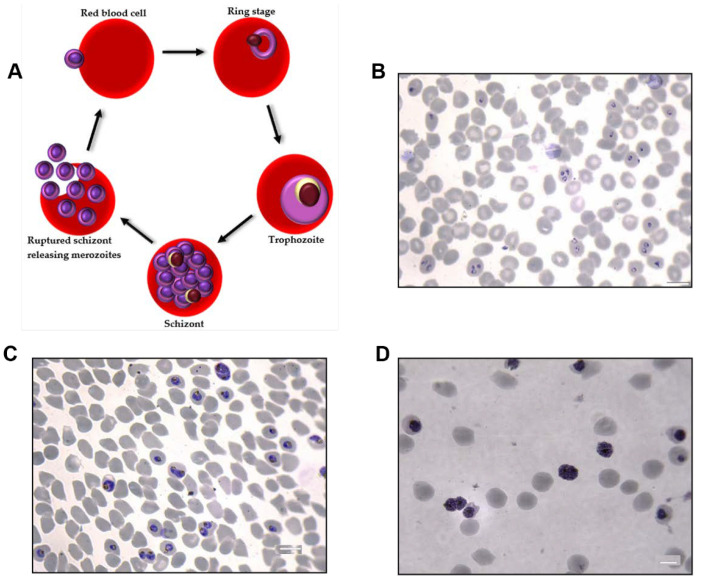
(**A**) The intraerythrocytic life cycle of malaria parasites. Ring, trophozoite and schizont stages last for approximately 24, 14 and 10 h, respectively. Parasite cultures were kept tightly synchronized to contain ≥85% of a specific blood stage. Conditioned culture media was harvested from cultures at 22-, 38- and 46-h post-invasion, to ensure the EVs present were released from (**B**), ring-, (**C**) trophozoite- or (**D**) schizont-iRBCs. Scale bar–10 µm.

In an in vitro culture, *P. falciparum* develops through the intraerythrocytic cycle as occurs in natural infection (Figure 1A). *P. falciparum* ring stages occupy, on average, a fifth of the intracellular space of RBCs. Trophozoites occupy a larger portion of the RBC cytoplasm and contain a dark mass of malaria pigment. Schizonts also contain the malaria pigment and may fill two-thirds or more of the iRBC. The schizont cytoplasm is segmented and contains 8–24 merozoites. Merozoites are released from iRBCs when the schizont ruptures at the end of the 48-h intraerythrocytic cycle. Each merozoite invades a new RBC and the cycle continues. The ring stages are observed up to 24 h post merozoite invasion. Trophozoites are observed between 22- and 38-h post invasion and the schizont stage lasts up to 10 h from 38 to 48 h post invasion.

The 3D7 strain of *P. falciparum* was grown in fresh human A + RBCs (up to 14 days old) in RPMI 1640 supplemented with 0.5% Albumax I, 10mM hypoxanthine, 1M NaOH and 50mg/mL gentamicin. Cultures were incubated at 37 °C with atmospheric conditions of 5% CO_2_, 3% O_2_ and 92% N. Uninfected RBC (uRBC) control cultures were maintained under identical conditions. *P. falciparum* cultures were maintained at parasitemias between 4–25% [29] and hematocrit ranging from 0.8–4.3%. The parasites were kept tightly synchronized using 5% sorbitol at the ring stage and 70% Percoll at the schizont stage (Figure 1B–D). 

### 2.3. EV Isolation

Conditioned culture medium (CCM) was harvested from synchronous cultures at 22 h, 38 h and 46 h post invasion for the isolation of *P. falciparum-*infected RBC EVs (*Pf*iRBC-EVs) from ring, trophozoite and schizont stage infected RBCs, respectively (Figure 1B–D). CCM was harvested from uRBC cultures every 48 h after which uRBC CCM was treated the same as iRBC CCM. For each sample, the starting CCM volume was 300 mL as this volume yielded the best results.

**Figure 2:**
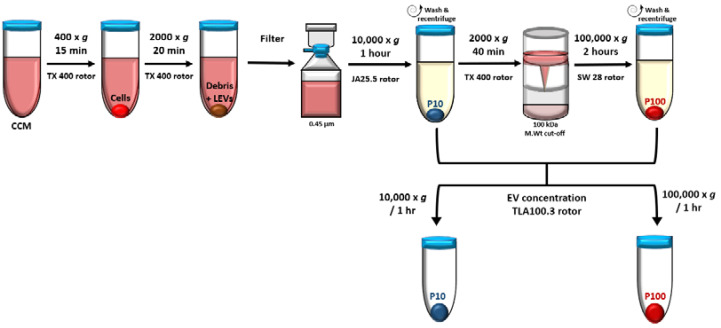
EVs were isolated by differential centrifugation including a filtration and concentration step. Medium speed centrifugation at 10,000× *g* and high-speed ultracentrifugation at 100,000× *g* was used to pellet EVs designated P10 and P100, respectively. CCM—conditioned culture media, LEV—large EVs.

EVs were isolated by differential centrifugation (Figure 2). Cultures were first centrifuged briefly for 5 min at 300× *g* at 25 °C to clear the CCM of cells which were returned to culture. All proceeding steps in the EV isolation procedure were performed at 4 °C; 300 mL of cleared, pooled CCM each from the ring, trophozoite, schizont, or uRBC cultures was further centrifuged at 400× *g* for 15 min and 2000× *g* for 20 min to completely remove any remaining cells, debris and large EVs. The clear supernatant was passed through a 0.45 μm pore PES vacuum filter after which the filtrate was centrifuged at 10,000× *g* for 1 h using a JA25.50 fixed angle rotor to obtain the 10K pellet henceforth designated P10 and portray EVs isolated at this stage. The supernatant was then concentrated in 100,000 Da MWCO Vivacell concentrators that were centrifuged at 2000× *g* for 40 min using a TX 400 rotor. 

The pooled concentrate (~10 mL) was diluted to 35 mL in PBS and centrifuged at 100,000× *g* for 2 h in an SW28 swinging bucket rotor to obtain the 100 K pellet. This pellet was designated P100 and portrays EVs isolated at this stage. P10 and P100 were washed in 30–35 mL of sterile PBS and recentrifuged at the same speed and duration used to isolate the respective pellets. Finally, EVs were concentrated in 1 mL PBS in a TLA100.3 rotor for 1 h at 10,000× *g* (14,000 rpm) for P10 and 100,000× *g* (43,000 rpm) for P100. EVs were resuspended in 50 µL sterile PBS and aliquoted for transmission electron microscopy (TEM), nanoparticle tracking analysis (NTA) and Western blot analysis (WBA). EVs were stored at −80 °C until needed.

### 2.4. Transmission Electron Microscopy

Frozen EVs were thawed at 4 °C and fixed in 2.5% glutaraldehyde in 0.1 M sodium-cacodylate buffer. EV preparations were deposited on formvar-carbon coated grids and negatively stained with 2% uranyl acetate solution. Grids were examined at 120 kV, with all images collected on an FEI Tecnai G2 Spirit Twin electron microscope located at the Facility for Electron Microscopy Research at McGill University.

### 2.5. Nanoparticle Tracking Analysis

The size distribution and concentration of vesicles were performed by NTA using the Nanosight NS300 (Malvern Panalytical) located at the Centre for Applied Nanomedicine (CAN) platform at the Research Institute of the McGill University Health Center. EVs were thawed at 4 °C and diluted 1 in 80 or 1 in 40 with PBS. Each sample was captured five times and the results were represented as the mean of all five captures.

### 2.6. SDS-PAGE and Western Blot Analysis

*P. falciparum* rings, trophozoites and schizonts were prepared by lysing stage-specific iRBCs with 0.01% saponin. Pelleted iRBCs from culture were washed in PBS to remove all culture media. The cells were then resuspended in up to 40 mL of 0.01% saponin and mixed vigorously till the turbidity of the mixture had visibly reduced indicating lysis of iRBC membranes. Released whole parasites in the suspension were pelleted by centrifugation at 2000× *g* followed by two washes in PBS.

RBC cytosol was obtained by hemolysis of uRBCs from culture in 5 mM sodium phosphate, followed by washing of the pellet three times in 5 mM sodium phosphate at 20,000× *g* for 15 min to obtain RBC ghosts devoid of cytosol [30]. uRBC ghosts and cytosol, EV pellets P10 and P100, as well as pellets of whole parasites from corresponding ring, trophozoite and schizont cultures, were lysed in RIPA containing protease inhibitor (Thermofisher Scientific; Waltham, MA, USA). The protein concentration in the lysates was measured using the Pierce^TM^ BCA protein assay kit (Thermofisher Scientific). All protocols were performed according to the manufacturer’s specifications.

Equivalent amounts of proteins of lysates were separated on Novex^TM^ 4–12% or 4–20% tris-glycine polyacrylamide gels (Invitrogen) under reducing conditions (anti-GPA, anti-*Pf*GRP78, anti-flotillin 2, anti-hemoglobin and anti-albumin) or non-reducing conditions (anti-CD63 and anti-CD81). The amount of protein loaded onto each gel (0.4 or 0.5 µg) was pre-determined by experiments to determine the linear range of detection of each antibody analyzed and for each sample, as previously described [31] (data not shown). Polyacrylamide gels were stained using a silver stain technique (ThermoFisher; Waltham, United States) to visualize the separated EV proteins. Separated proteins were transferred to PVDF membranes (Millipore; Waltham, MA, USA), which were incubated with primary antibody overnight at 4 °C, followed by a 1-h incubation with secondary antibody at room temperature. Proteins were detected on PVDF membranes following treatment with SuperSignal West Pico PLUS chemiluminescent substrate (Thermo Scientific) and imaging with the Bio-Rad Chemidoc MP Imaging System. 

Total protein staining was used as an internal loading control [31] and membranes were stained using colloidal silver as previously described [32]. The signal intensity of bands was quantified by ImageJ (v1.53o) software.

## 3. Results and Discussion

### 3.1. Characterization of Single EVs from P. falciparum-Infected RBCs 

**Figure 3:**
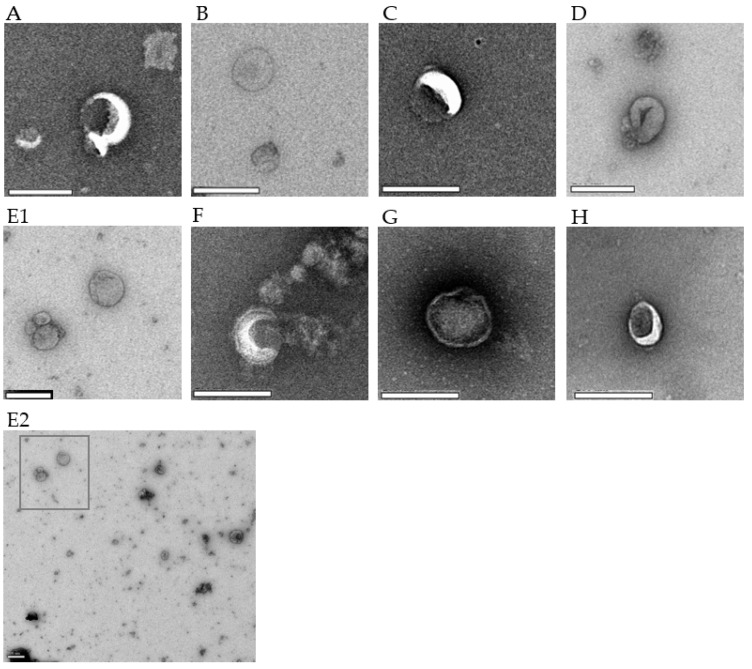
TEM micrographs of *P. falciparum* iRBC EVs and uRBC EVs. Each image is representative of 4–6 captures. (**A**) Ring-P10, 18,500× (**B**) Ring-P100, 18,500× (**C**) Trophozoite-P10, 23,000× (**D**) Trophozoite-P100, 18,500× (**E1**) Schizont-P10, 13,000× (**E2**) A representative wide-field image showing heterogeneity of EVs; E1 was taken from this field (**F**) Schizont-P100, 23,000× (**G**) uRBC-P10, 23,000× (**H**) uRBC-P100, 23,000×. Scale bar–200 µm.

All EV preparations of iRBCs and uRBCs were analyzed using TEM and NTA to best evaluate the consistency of the isolation process. TEM revealed membrane-bound vesicles with a size range of 50–300 nm (Figure 3), where some vesicles appeared more electron dense than others across the stage-specific iRBC EVs.

**Figure 4:**
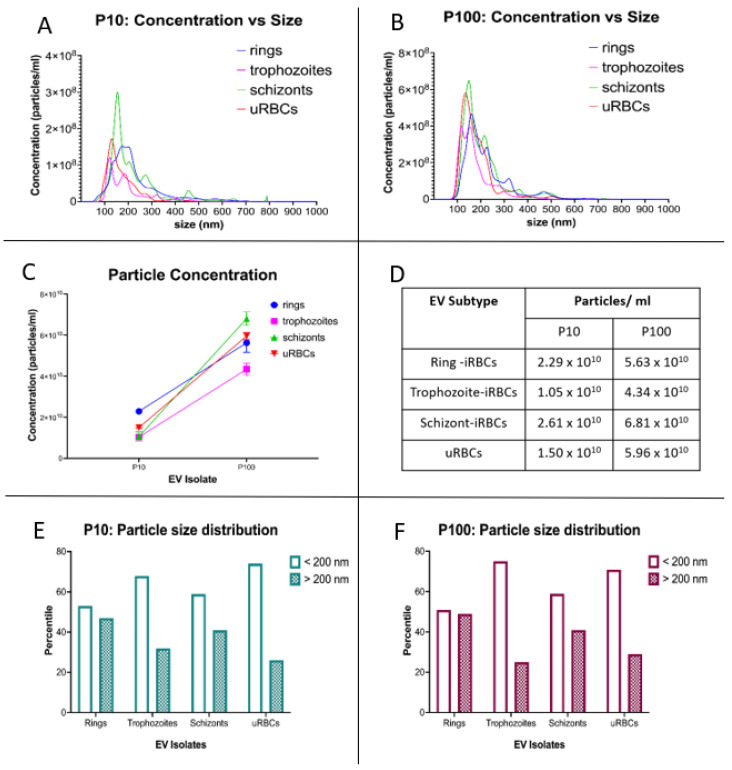
NTA of *P. falciparum* iRBC EVs and uRBC EVs. Particle concentration and size distribution for (**A**) P10 and (**B**) P100. (**C**,**D**) Analysis of particle concentration showed that for iRBCs and uRBCs, a significantly higher proportion of secreted EVs is contained in P100. (**E**) P10 & (**F**) P100 EV preparations contain more EVs that are <200 nm in diameter. iRBCs—infected RBCs, uRBCs—uninfected RBCs.

The size distribution of EVs was corroborated by NTA, which showed the majority of P10 and P100 EV samples to be between 100 and 300 nm in diameter (Figure 4A,B). As shown in Figure 4A, the peak diameter for P10 EVs slightly varied across the different populations as follows: ring-iRBCs–179.3 nm, trophozoite-iRBCs–121.8 nm, schizont-iRBCs–154.1 nm and uRBCs–129.6 nm. The peak diameter for P100 EVs (Figure 4B) was as follows: ring-iRBCs–159.7 nm, trophozoite-iRBCs–116.9 nm, schizont-iRBCs–148.6 nm and uRBCs–134.6 nm. For both P10 and P100 EV isolates, the largest to smallest EVs were rings > schizonts > uRBCs > trophozoites. These modal sizes all correspond to the physical characteristic of small EVs, which are determined to be <200 nm [20]. 

By comparing the concentration of released vesicles, we observed that P100 was 2.5–4 times greater than P10 in all samples (Figure 4C,D). While the number of EVs smaller than 200 nm in diameter was only slightly higher in the ring-iRBCs for both P10 and P100, the majority of trophozoite- and schizont-iRBC P10 and P100 EVs were smaller than 200 nm (Figure 4E,F).

### 3.2. Analysis of EV Protein Marker Profiles of Stage-Specific P. falciparum iRBC EVs

To further demonstrate the presence of EVs and determine their nature, we analyzed the malaria-derived EV preparations and uRBC EVs for CD63, CD81, glycophorin A (GPA), flotillin 2, *Pf*GRP78 as well as albumin and hemoglobin by immunoblotting. For all immunoblots performed in this study, a non-conditioned medium (NCM) was included as a control to verify that EV markers were solely associated with isolated EVs [33]. 

**Figure 5:**
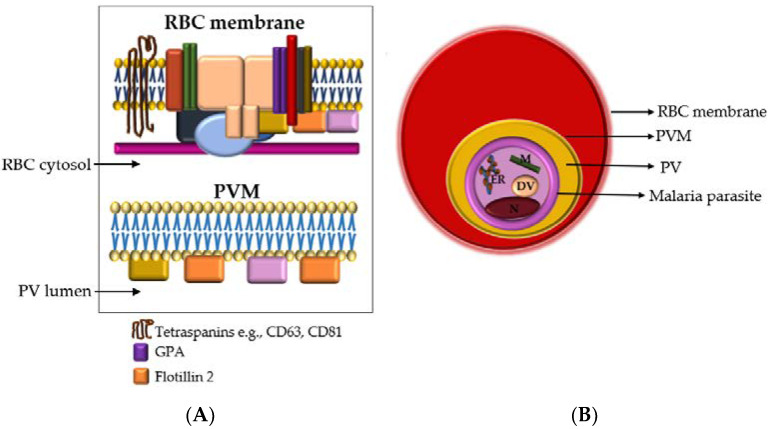
(**A**) Cross-section of the RBC membrane (**top**) and the malaria parasitophorous vacuolar membrane (PVM) (**bottom**). The parasite resides in the RBC cytosol. The parasitophorous vacuole (PV) lumen is the narrow space between the PVM and the parasite membrane (PM). Glycophorin A (GPA) is the major integral protein of the RBC membrane and forms part of the RBC membrane multiprotein complex. Other proteins in this complex that are not specified here are represented by the various shapes and colors and include band 3, spectrin, actin, etc. The malaria parasite PVM is rich in flotillins (1 & 2) but also contains other lipid raft proteins, such as CD55 and CD59 (represented by yellow and pink) (**B**) Within an infected RBC, the malaria parasite resides inside a PV that is encased in a parasitophorous vacuolar membrane. EVs secreted by the parasite would need to pass three membranes: the parasite plasma membrane, the PVM and the RBC membrane. Among the malaria parasite organelles shown are the endoplasmic reticulum (ER) that expresses *Pf*GRP78, mitochondria (M), digestive vacuole (DV), and nucleus (N). RBC—red blood cell, PVM—parasitophorous vacuolar membrane, PV—parasitophorous vacuole.

CD63, CD81 and GPA are transmembrane proteins, while flotillin 2 is a cytosolic membrane-associated protein found in lipid rafts (Figure 5A). The presence of a cytosolic membrane-associated protein and transmembrane protein in an isolate implies that it contains intracellular elements enclosed within lipid bilayers which is the characteristic nature of EVs [20]. *Pf*GRP78 is the *Plasmodium* spp. orthologue of the endoplasmic reticulum binding immunoglobulin protein (BiP) (Figure 5B). *Pf*GRP78 is either absent from or not enriched in EVs [20]. 

#### 3.2.1. Albumin, Hemoglobin and PfGRP78

**Figure 6:**
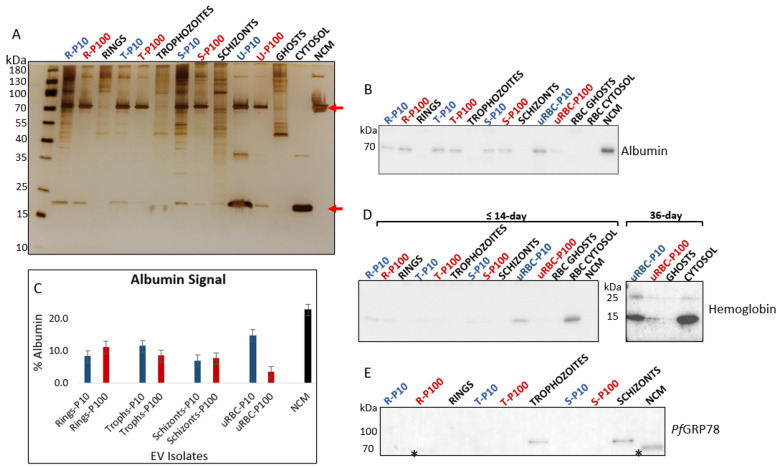
(**A**) SDS-PAGE and silver staining of EVs and cell lysates revealed distinct protein separation profiles. Top red arrow is albumin (70 kDa), and bottom red arrow is hemoglobin (16 kDa). (**B**) Immunoblot of albumin. Albumin present in culture medium co-isolates with EVs. NCM was included as a control to assess the extent of albumin depletion from EV isolates. No albumin was present in cell lysates. (**C**) Densitometric analysis of albumin in EV isolates. (**D**) Hemoglobin is more abundant in EVs of aging RBCs and almost undetectable in the iRBC EVs from pooled cultures of RBCs ≤ 14 days. Immunoblot shows an increase in hemoglobin content from ≤14-day to 36-day old RBCs. (**E**) *Pf*GRP78 (78 kDa) is absent from all malaria derived EVs and can only be seen in trophozoite and schizont lysates. Anti-*Pf*GRP78 cross-reacts with albumin in samples (lower band, marked with asterisk). P10 EVs–blue, P100 EVs–red, cell lysates and controls–black. R—rings, T—trophozoites, S—schizonts, uRBC—uninfected RBCs, NCM—non-conditioned media.

EVs were analyzed for albumin and hemoglobin to assess the quality of the preparations. Albumin, more specifically, was used as a purity control for EV isolation [20]. Albumin was detected as a co-isolated protein in the silver-stained polyacrylamide gel (Figure 6A) and immunoblot (Figure 6B) at ~70 kDa. The densitometric analysis demonstrated depletion of albumin contamination from EV isolates (Figure 6C). 

Hemoglobin, the predominant cytosolic protein of RBCs, is mainly lost by vesiculation that can occur: through the physiological aging process of RBCs [34], during storage under blood bank conditions [35,36], and in vitro by treating RBCs with the cation ionophore A23187 to induce vesicle release [37,38]. Studies have shown that beyond two weeks of storage, RBCs release significantly more EVs; these EVs characteristically show signs of degeneration and contain higher amounts of protein (including hemoglobin) that proportionally increase with the age of the RBC [35,36]. For parasite cultures in this study, mature RBCs not older than two weeks were used for EV isolation. In addition, old RBCs do not support the healthy growth of malaria parasites, particularly in cultures of high parasitemia [29], as was required in this study.

Hemoglobin was present only in trace amounts in the parasite-infected RBC EVs–P10 and P100, as detected on the silver-stained polyacrylamide gel (Figure 6A) and the immunoblot (Figure 6D). This suggests that hemoglobin is not a major component of *P. falciparum*-infected RBC-derived EVs. During the intraerythrocytic cycle, the malaria parasite ingests and digests immense amounts of hemoglobin to support its development [39,40]. Therefore, it is unlikely that EVs secreted by the parasite carries large quantities of hemoglobin. It is important to note, however, that EVs released from iRBCs most likely comprise a mixed population of parasite- and RBC-derived EVs, the latter of which may contain larger quantities of hemoglobin.

To attest to the presence of hemoglobin in aging RBC EVs, we performed a hemoglobin immunoblot of 36-day old RBCs and observed a greater abundance of hemoglobin in both P10 and P100 EVs compared to the EVs from the 14-day old RBCs (Figure 6D). In addition to the 16 kDa monomer, a 28 kDa hemoglobin dimer was detected in the 36-day old RBC-EVs and RBC cytosol. This dimer is a characteristic storage lesion of aging RBCs [35]. The absence of this dimer from iRBC EVs is a useful negative marker in malaria EV research when determining the suitability of host RBCs for culture and quality of a malaria-EV preparation.

*Pf*GRP78 (also known as BiP), a chaperone protein of the endoplasmic reticulum, has no association with the central components of EV biogenesis, i.e., the plasma membrane and endosomes. As such *Pf*GRP78, in theory, is not expected to be found in small EVs (specifically EVs smaller than 200 nm in diameter) but may be present in any given EV subtype due to active or passive cargo loading [20]. In this study, no *Pf*GRP78 was detected in parasite iRBC EVs and was only found in whole trophozoite and schizont lysates (Figure 6E). 

The analysis of proteins localized to intracellular compartments of the malaria parasite (i.e., not secreted to the RBC cytosol or membrane) is important in elucidating malaria EV biogenesis. The malaria parasite inside an infected RBC resides within a parasitophorous vacuole that is enclosed in a membrane formed from the RBC membrane and parasite material, i.e., the parasitophorous vacuolar membrane (PVM) [23] (Figure 5B). EVs containing even trace amounts of *Pf*GRP78, or any other parasite-specific non-secretory proteins, would have to pass through at least three membranes–the parasite membrane, parasitophorous vacuolar membrane and, RBC membrane before being released from the infected RBC (Figure 5B). This would suggest a coordinated and complex EV biogenetic pathway directed by the malaria parasite. Further studies are underway to better elucidate this pathway.

#### 3.2.2. Tetraspanins: CD63 and CD81

**Figure 7:**
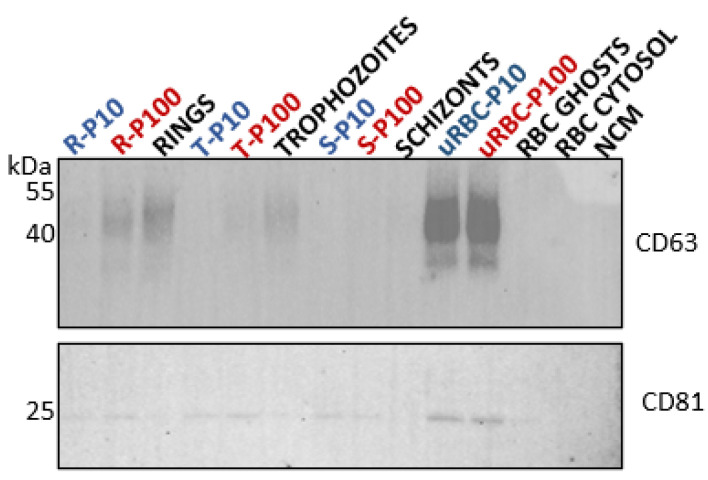
Western blot analysis of tetraspanins in EVs (blue and red) and lysates of parent cells from which the EVs were released (black). Tetraspanins CD63 (**top**, 40–50 kDa) and CD81 (**bottom**, 25 kDa) serve as general EV markers. R—rings, T—trophozoites, S—schizonts, uRBC—uninfected RBCs, NCM—nonconditioned media.

CD63 is a member of the tetraspanin family that has been reported to play key roles in the biogenesis of EVs. It is a transmembrane protein (Figure 5A) that is detected in EVs of endosomal and plasma membrane origin [41]. CD63 was heavily abundant in uRBC EVs (P10 and P100). Considering the conspicuous absence of CD63 from the RBC membrane (ghosts) in our study (Figure 7) and the absence of endosomal machinery in mature RBCs, the source of CD63 in the uRBC EVs is unclear. Unlike the RBC ghost membranes and cytosol, CD63 was detected in the *P. falciparum* ring and trophozoite lysates, which represent the parent cells from which the respective ring- and trophozoite-iRBC EVs were isolated.

The amount of CD63 appeared to diminish as the parasite matures from rings to trophozoites, with no detectable CD63 in the schizont cell lysate. This trend was observed as well for the iRBC P100 EVs with CD63 being present in ring-infected P100, to a lesser degree in trophozoite-infected P100 EVs, and undetectable in schizont-infected P100. CD63 was not detected in P10 EVs of either ring-, trophozoite-, or schizont-stages. 

Like CD63, tetraspanin CD81 is a transmembrane protein (Figure 5A) that is detected in EVs of endosomal and plasma membrane origin [41]. CD81 was detected in trace amounts in RBC ghosts as well as in ring and trophozoite cell lysates but not in the schizont cell lysate. Compared to the cell lysates, however, CD81 was detected in all EV populations, i.e., ring-, trophozoite-, and schizont-infected P10 and P100 EVs as well as uRBC P10 and P100 EVs (Figure 7). 

The CD63 and CD81 present in the parasite iRBC EVs may be a carry-over from the host RBCs, as a study of the phylogenetic analysis of tetraspanins reported none to be found in the genome of *P. falciparum* [42]. Nevertheless, the differential expression of these tetraspanins across ring-, trophozoite-, and schizont-infected RBC P10 and P100 EV populations (Table 1) suggests distinct biogenetic pathways that are influenced by the blood stage of the parasite, and which results in the secretion of different EV subtypes per blood stage that can be successfully separated by differential centrifugation, according to their pelleting properties. A recent study has suggested that CD81 rich EVs are more likely to be of plasma membrane origin while CD63 rich EVs are likely to be of endosomal origin [43]. Although neither of these EV markers were enriched in these EVs, it is important to investigate their trafficking and cellular location in RBCs and malaria iRBCs to gain insights into how parasite maturation in iRBCs influences EV biogenesis and release. Immunofluorescence assays are underway to explore this.

#### 3.2.3. Glycophorin A (CD235a)

**Figure 8:**
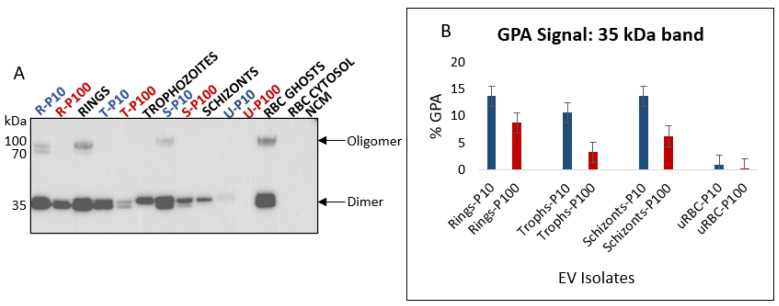
(**A**) Western blot analysis of GPA in EVs (blue and red) and lysates of parent cells from which the EVs were released (black). GPA is a transmembrane protein and an RBC specific EV marker; observed molecular weights are a 35 kDa dimer and 90 kDa oligomer. Lower molecular weight cleavage product can be observed for both the dimer and oligomer. (**B**) Densitometric analysis of 35 kDa GPA dimer in EVs. GPA is more enriched in iRBC P10 than iRBC P100 EVs. All iRBC EVs have significantly more GPA than uRBC EVs; P10 EVs–blue, P100 EVs–red, cell lysates and controls–black. R—rings, T—trophozoites, S—schizonts, U—uninfected, NCM—nonconditioned media.

Glycophorin A (GPA) is a sialoglycoprotein and is the major integral membrane protein of RBCs (Figure 5A). GPA is the designated EV marker for RBCs [20] and was detected as a 35 kDa dimer in all EV populations except for uRBC P100 EVs (Figure 8A) but was present when greater amounts of uRBC P100 EV protein were loaded (unpublished data). The lower molecular weight band of GPA was consistently present in greater proportions in P10 compared to P100 across all stage-specific infected RBC-derived EVs (Figure 8B). This is suggestive of differential sorting of GPA into the various EV subtypes. Furthermore, significantly greater amounts of GPA in EVs from iRBCs compared to uRBCs reflects the significant effect of *P. falciparum* infection on RBC EV biogenesis. This is also an indication of a distinct biogenetic pathway of iRBC-derived EVs, and cargo-loading compared to uRBC-derived EVs.

In addition to the 35 kDa dimer, a high molecular weight (~90 kDa) oligomer was observed for the ring-, and schizont-infected RBC P10 EVs, as well as ring lysates and RBC ghosts. Oligomerization of GPA in lipid membranes is a highly specific process that involves interactions between different domains [44]. Whether the presence of this high molecular weight GPA oligomer coupled with the absence of CD63 and the presence of CD81 in these P10 EVs (Table 1) is indicative of the selective isolation of plasma membrane budding EVs is uncertain and requires further investigation.

#### 3.2.4. Flotillin 2

**Figure 9:**
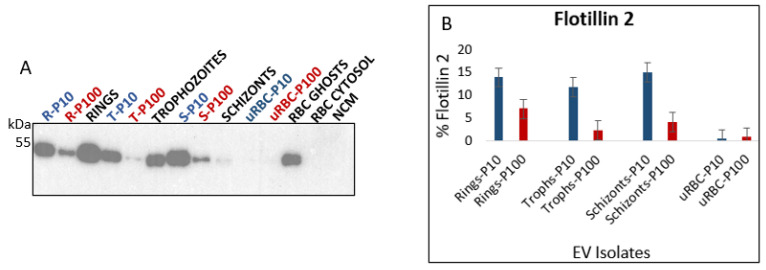
(**A**) Western blot analysis of flotillin 2 in EVs (blue and red) and lysates of parent cells from which the EVs were released (black). Flotillin 2 (49 kDa) is a cytosolic membrane-associated protein that is commonly found in EVs. (**B**) Densitometric analysis of flotillin 2. Flotillin 2 is enriched in iRBC P10 compared to iRBC P100 EVs. All iRBC EVs have significantly more flotillin 2 than uRBC EVs. P10 EVs–blue, P100 EVs–red, cell lysates and controls–black. R—rings, T—trophozoites, S—schizonts, U—uninfected, NCM—nonconditioned media.

The RBC membrane bilipid layer contains lipid rafts [45], which in turn are key players in vesicle formation [46]. There are several proteins on the cytosolic side of the RBC membrane that are associated with these lipid rafts. One such protein is flotillin 2 (Figure 5A, top). Malaria parasitophorous vacuolar membrane, like RBC membrane lipid rafts, is rich in flotillins (1 and 2) that are incorporated into it from the host RBC membrane during parasite invasion [47,48] (Figure 5A, bottom). The presence of lipid rafts in all EV isolates was demonstrated by detecting flotillin 2 (Figure 9A). Studies have shown that flotillin 2 and other lipid raft proteins, including flotillin 1, stomatin, CD55 and CD59 are differentially expressed in RBC EVs depending on the fashion in which they are produced [27,34,35,37,49]. We found flotillin 2 to be enriched in iRBC EVs to a large extent compared to uRBC EVs (Figure 9B). This was also observed for GPA (Figure 8) and again has implications for malaria infection on altered EV biogenesis and cargo loading in iRBCs.

Flotillin 2 was significantly more abundant in P10 ring-, trophozoite-, and schizont-iRBC EVs compared to the P100 EVs. Unlike GPA, however, the amount of flotillin 2 in trophozoite-iRBC P100 EVs was very low. This suggests that, while flotillin 2 may be a suitable marker for CD63-negative malaria derived P10 EVs, it is unlikely to be deemed a suitable adjunct marker for CD63-positive malaria derived P100 EVs (Figure 7 and Table 1).

To gain insights into the contribution and nature of lipid rafts in vesiculation in *P. falciparum* iRBCs, it is important to comparatively analyze multiple lipid raft-associated proteins and demonstrate the presence and organization of the lipid raft regions on these EVs. These experiments are planned.

## 4. Conclusions

Using an optimized protocol, we have isolated and characterized malaria derived EVs from in vitro *P. falciparum* cultures. A significantly higher proportion of EVs was pelleted at 100,000× *g* (P100) than at 10,000× *g* (P10). For trophozoite and schizont-iRBCs, a large proportion of EVs in both fractions (P10 and P100) were smaller than 200 nm in diameter, while for ring-iRBCs, there was only a small difference between P10 and P100 EVs smaller or larger than 200 nm. Despite the variation in particle size distribution, EVs were separated by differential centrifugation into distinct subpopulations. CD63 was only detected in P100 EVs for RBCs infected with the ring and trophozoite blood stages of the parasite, however, all EV populations expressed CD81. Oligomers of GPA were observed only in P10 EVs, while the GPA dimer and flotillin 2 were significantly more abundant in P10 than in P100 EVs for the ring-, trophozoite-, and schizont-iRBCs. Among all three stages of *P. falciparum*, GPA and flotillin 2 were least abundant in trophozoite-iRBC P100 EVs, with flotillin 2 being almost undetectable. The profile of iRBC EVs differed from that of uRBC EVs for all proteins analyzed.

It is evident from our preliminary findings that the blood stage of *P. falciparum* influences the nature and composition of secreted EVs from iRBCs. Investigating the localization and trafficking of tetraspanins in infected and uninfected RBCs is essential to understanding malaria EV biogenesis. We identify the need to extensively analyze different RBC membrane proteins, RBC membrane-associated proteins and specific *P. falciparum* proteins to fully characterize blood-stage specific iRBC EVs and elucidate their biology. Characterization of EVs in malaria infection is an invaluable step towards the discovery of malaria EV markers, identifying their unique and/or shared functions as well as exploring their biomedical applications.

## Figures and Tables

**Table 1:** Summary of protein profile of malaria-derived EVs defined by the presence or absence of EV markers.

EV Subtype		CD63	CD81	GPA
Ring-iRBC	P10	-	+	++
	P100	+	+	++
Trophozoite-iRBC	P10	-	+	++
	P100	+	+	+
Schizont-iRBC	P10	-	+	++
	P100	-	+	++
uRBC	P10	++	+	+
	P100	++	+	-

++ enriched, + detected but not enriched, - not detected; iRBCs—infected RBCs, uR-BCs—uninfected RBCs.

## Data Availability

Not applicable.
